# Cardiac mitochondria exhibit dynamic functional clustering

**DOI:** 10.3389/fphys.2014.00329

**Published:** 2014-09-02

**Authors:** Felix T. Kurz, Miguel A. Aon, Brian O'Rourke, Antonis A. Armoundas

**Affiliations:** ^1^Department of Neuroradiology, Heidelberg University HospitalHeidelberg, Germany; ^2^Cardiovascular Research Center, Harvard Medical School, Massachusetts General HospitalCharlestown, MA, USA; ^3^Division of Cardiology, Department of Medicine, Johns Hopkins UniversityBaltimore, MD, USA

**Keywords:** mitochondrial clustering, mitochondrial oscillator, functional connectivity, wavelets, cardiac myocyte

## Abstract

Multi-oscillatory behavior of mitochondrial inner membrane potential ΔΨ_*m*_ in self-organized cardiac mitochondrial networks can be triggered by metabolic or oxidative stress. Spatio-temporal analyses of cardiac mitochondrial networks have shown that mitochondria are heterogeneously organized in synchronously oscillating clusters in which the mean cluster frequency and size are inversely correlated, thus suggesting a modulation of cluster frequency through local inter-mitochondrial coupling. In this study, we propose a method to examine the mitochondrial network's topology through quantification of its dynamic local clustering coefficients. Individual mitochondrial ΔΨ_*m*_ oscillation signals were identified for each cardiac myocyte and cross-correlated with all network mitochondria using previously described methods (Kurz et al., 2010a). Time-varying inter-mitochondrial connectivity, defined for mitochondria in the whole network whose signals are at least 90% correlated at any given time point, allowed considering functional local clustering coefficients. It is shown that mitochondrial clustering in isolated cardiac myocytes changes dynamically and is significantly higher than for random mitochondrial networks that are constructed using the Erdös–Rényi model based on the same sets of vertices. The network's time-averaged clustering coefficient for cardiac myocytes was found to be 0.500 ± 0.051 (*N* = 9) vs. 0.061 ± 0.020 for random networks, respectively. Our results demonstrate that cardiac mitochondria constitute a network with dynamically connected constituents whose topological organization is prone to clustering. Cluster partitioning in networks of coupled oscillators has been observed in scale-free and chaotic systems and is therefore in good agreement with previous models of cardiac mitochondrial networks.

## Introduction

The mitochondrial network in cardiac myocytes consists of highly organized and densely packed mitochondria whose inner membrane potential ΔΨ*_m_* can be triggered to enter several cycles of de-and re-polarizations by numerous stressors such as oxidative or metabolic stress (see Aon et al., 2008a for a review). These oscillations can be strictly localized in the form of transient single mitochondrial depolarizations (Nivala et al., 2011), individual or clustered mitochondrial ΔΨ*_m_* oscillations (Romashko et al., 1998; Kurz et al., 2010a) with clusters that can span the whole myocyte (Aon et al., 2004). Recruiting neighboring network mitochondria into an initial synchronized nucleus of a few mitochondrial oscillators has been described to be a fundamental process for global network synchronization (Strogatz, 2000, 2003; Aon et al., 2008b). During this process, a mitochondrial cluster can reach a critical size (sometimes referred to as “mitochondrial criticality” Aon et al., 2004, 2006) where mitochondria spontaneously self-synchronize, as in a phase transition. So far, investigations strongly support the fact that ROS-induced ROS release is a key player in such inter-mitochondrial communication or coupling (Zorov et al., 2006; Zhou et al., 2010; Nivala et al., 2011).

Recently, wavelet-based analysis tools have been developed to examine the mitochondrial network's spatio-temporal behavior under pathophysiological conditions (Kurz et al., 2010a,b); dynamic frequencies could be allocated to individual mitochondria and clusters of mitochondria with similar frequencies were identified that allowed for a quantitative characterization of the cluster's network properties. However, the network's topology (or connectivity properties) as opposed to its architectural organization (see Aon and Cortassa, 2012 for a review) has not yet been investigated quantitatively in terms of its clustering properties. Mitochondria in cardiac myocytes serve as the main energy supplier and modulator of the myocyte's mechanical and electrical processes, but are also modulated by the latter; therefore, the mitochondrial network's topological heterarchy becomes increasingly complex and non-linear (Yates, 1993). The functionality of an individual mitochondrial network node, though, can in part be characterized through its connectedness with other network nodes, (cf. Passingham et al., 2002), a result of the interplay of the entire complex mitochondrial network as an integrated system. The clustering coefficient can be used as a measure of the network's robustness toward the functional deletion of single mitochondria or the network's efficiency to communicate (metabolic or other) information.

The present work investigates the presence of functional (dynamical) connectedness in the form of clustering of mitochondrial networks in isolated cardiac myocytes in comparison with clustering in random networks based on the Erdös–Rényi model. Functional network clustering is subsequently related to the network's spatio-temporal properties of the major cluster of mitochondria with similar frequencies.

## Materials and methods

### Experimental methods

All experiments were carried out on freshly isolated adult guinea pig ventricular myocytes at 37°C following protocols that were previously described (O'Rourke et al., 1994) with approval from the Johns Hopkins University Animal Care and Use Committee and in accordance with guidelines established in the *Guide for the Care and Use of Laboratory Animals*, published by the National Institutes of Health (NIH Publication No. 85-23, Revised 1996).

In brief, cardiac myocytes were perfused with Tyrode solution (pH 7.5) containing 1 mM Ca^2+^ in the presence of 10 mM glucose and oscillations were triggered with a localized (5 × 5 μm) laser flash, as previously described (Aon et al., 2003). Mitochondrial inner membrane potential ΔΨ*_m_* was monitored with the cationic potentiometric fluorescent dye tetramethylrhodamine methyl ester (TMRE) and images were recorded with a two-photon laser-scanning microscope (MRC-1024MP, Bio-Rad) with excitation at 740 nm (Tsunami Ti:Sa laser, Spectra-Physics) and red emission of TMRE was collected at 605 nm using a band pass filter 578–630 nm (Aon et al., 2003).

### Selection and processing of individual mitochondrial TMRE signals

As detailed before (Kurz et al., 2010a,b), TMRE signals of individual mitochondria were extracted from planar images of isolated cardiac myocytes recorded at a rate of *dt* by manually applying a grid template on a pixel-by-pixel basis to the averaged image of *n* subsequent images in time starting at the onset of TMRE oscillations. Parameter *n* was chosen such that *n* · *dt* was smaller or equal to the smallest period of all TMRE oscillations. Only myocytes with no shifts in the z-direction were included and shifts in the x and y-directions were corrected by moving the template grid accordingly.

The subsequent spatio-temporal signal analysis was conducted using the wavelet transform, correlation and coherence analysis as previously described in detail (Kurz et al., 2010a) and outlined below.

### Wavelet analysis

With no prior knowledge of whether mitochondrial oscillations are stationary, wavelet analysis has been used to probe the dynamically changing frequencies of cardiac mitochondria (Kurz et al., 2010a). In this study, the same wavelet analysis was utilized: for each mitochondrion's TMRE signal, Morlet wavelets were taken where spacing between scales was set to *dj* = 0.1 and the smallest scale of the wavelets was chosen as *s*_0_ = 4*dt*, signifying the smallest possible period for the detection of one oscillation. The total number of scales was determined as *j*_1_ = log_2_ (*N*/*s*_0_)/*dj* + 1 with *N* being the total number of the recorded images per cell, thus resulting in scales that range from *s*_0_ to *s*_0_ 2^(*j*_1_ −1)*dj*^ and each scale having *dj* suboctaves. To determine cutoff frequencies, the longest period *T* of a synchronized oscillation of one cell was identified to determine the minimum cutoff frequency as ν_min_ = 1/1.1*T* and, similarly, the maximum cutoff frequency at ν_max_ = 1/*s*_0_. Power lineplots between ν_min_ and ν_max_ were interpolated for every scale with segments of 0.1 mHz and the maximum power for the interpolated plots was determined to eventually obtain maximal scale frequencies at each time point for each mitochondrion.

**Figure 1:**
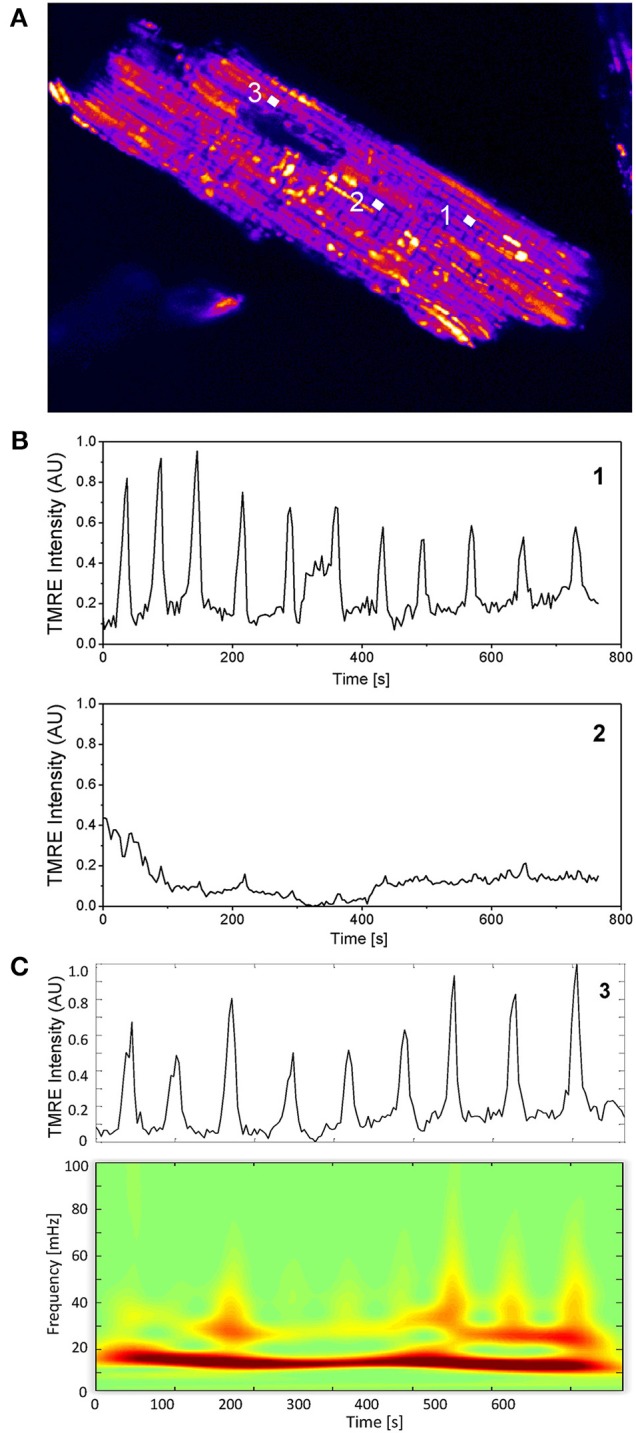
**Single mitochondrial signals and wavelet analysis. (A)** Mitochondria in cardiac myocytes are densely packed and their individual TMRE signal can be extracted as previously described (Kurz et al., 2010a,b). **(B)** Two mitochondrial signals with different oscillatory patterns from different locations within the myocyte are shown. **(C)** Absolute squared wavelet transform over frequency and time of an oscillating mitochondrion. The major frequency component varies between 15 and 20 mHz.

In Figure 1A, three random mitochondria labeled 1, 2, and 3 from an isolated cardiac myocyte are chosen to illustrate individual mitochondrial TMRE signal behavior. Evidently, only mitochondria 1 and 3 show marked oscillatory behavior over time whereas mitochondrion 2 is non-oscillating (see Figures 1B,C). Figure 1C shows the absolute squared wavelet transform of mitochondrion 3 over frequency and time. It can be seen that the main frequency component, depicted by the dark red color, varies between 15 and 20 mHz during the recording. This frequency component corresponds to the time interval between oscillation peaks and troughs observed in the upper panel of Figure 1C, that is approximately between 50 and 65 s.

### Selection of mitochondria belonging to a major cluster

By obtaining frequency histograms of all mitochondria for each time point *t*, the maximum peak of each histogram was identified and other peaks were considered significant if their histogram amplitude was above 10% of the respective maximum peak (see also Figure 2A in Kurz et al., 2010a). If the mean TMRE signal of all mitochondria corresponding to the maximum peak had a cross correlation of 95% or higher with the mean TMRE signal of mitochondria in an adjacent peak, the latter was incorporated in the maximum peak and the procedure repeated with the next adjacent peak until the correlation dropped below 95%. The respective signal cross correlation was determined over a running window *T_w_* = 1.1*T* with center at time-point *t*. In the resulting maximum peak at each *t*, all mitochondria in that cluster were identified and their mean TMRE signal in *T_w_* was cross-correlated with that of each mitochondrion in the myocyte that did not belong to the cluster. Again, if the correlation coefficient was 95% or higher, the individual mitochondria were incorporated into the cluster, thus yielding the major cluster of mitochondria that are highly correlated at time *t*. The normalized dynamic area of the major cluster was taken as the quotient of the total dynamic cluster pixel count and the total myocyte pixel-count.

### Coherence analysis

Temporal properties of major cluster mitochondria were examined by considering the average coherence of each cluster mitochondrion's TMRE signal with all of its nearest neighbors. Coherence values range between one and zero, indicating whether two signals oscillate synchronously at each frequency or not, respectively. Using a running window *T_w_*, a fixed Fast Fourier Transform length of 2^11^ *dt*, a frequency range for each myocyte of 0–100 mHz and its division into (2^11^/2) + 1 segments (each segment therefore corresponding to ≈0.1 mHz), the coherence was determined between each mitochondrion and its nearest neighbors for each time point and the mean time-resolved coherence over all nearest neighbors was calculated. Consequently, the average coherence for each major mitochondrial cluster at each time point was obtained. To compare myocytes with unequal duration of recordings, the duration of oscillation of each cardiac cell was set to 1.

### Mitochondrial cluster amplitude

For the mean TMRE signal of the major cluster, all peaks and troughs were manually identified and the respective peak-trough amplitudes and peak-trough time differences were determined (see also Figure 4 in Kurz et al., 2010a). Normalization of peak-trough amplitudes was achieved by dividing the amplitudes through the respective maximum amplitude.

### Local clustering coefficient

In graph theory, clustering describes the functional topology of a network by quantifying the degree to which a set of network vertices (or constituents) resembles the graph-theoretical concept of a clique (i.e., a set of vertices in which every two vertices are connected to each other). Considering a single network vertex *m*, the fraction of those network vertices that are (undirectedly) connected to vertex *m*, with respect to the maximum number of topological neighbors of vertex *m*, are described by the clustering coefficient *C_m_*. Evidently, if vertex *m* has *m_N_* topological neighbors, then the maximum number of possible undirected links among these neighbors is *m_N_* (*m_N_* −1)/2. With *L_m_* being the number of undirected links between the neighbors of mitochondrion *m*, local clustering *C_m_* is hence defined as *C_m_* = 2*L_m_*/ (*m_N_* (*m_N_* −1)). According to Watts and Strogatz (1998), the mean clustering coefficient of the whole complex network *C* is determined by the arithmetic mean of all local clustering coefficients *C_m_*: *C* = (1/*M*)∑ *_m_C_m_* with *M* being the number of network mitochondria.

### Functional connectivity

To determine the “functional” connectedness of the mitochondrial network, it is essential to determine the correlation coefficients between mitochondrial TMRE signals for each pair of mitochondria within the cardiomyocytes. For that matter, we followed a procedure by Eguiluz et al. (2005) to extract functional complex biological networks. This procedure abides by the recently introduced notion of the “spanning cluster” of oscillating mitochondria which stretches over the whole myocyte and does not necessarily involve all mitochondria (Aon et al., 2004, 2008a) as well as the concept of the dynamically changing major cluster of oscillating mitochondria described above (Kurz et al., 2010a). Two mitochondria were defined as being functionally connected at time *t* when the correlation coefficient of their TMRE signals in the time window *T_w_* around *t* was higher than a fixed cutoff value (see also **Figure 3A** in the results section). Consequently, clustering coefficients were determined for every individual mitochondrion and, for each considered myocyte (*N* = 9), mean clustering coefficients *C*(*t*) (i.e., averaged over all mitochondria at time *t* as in **Figure 3** or averaged over all major cluster mitochondria as in **Figure 4**) were determined for each point in time.

### Random network

For each myocyte, a random network was constructed that included the same number of mitochondria with the corresponding myocyte, using the Erdös–Rényi model (Erdös and Rényi, 1960). If *D_m_* (*t*) presents the number of links of mitochondrion *m* to its topological neighbors at time *t* (thus representing the dynamic degree of *m*), then each pair of mitochondria is connected with a dynamic probability *p*(*t*) = ∑*_m_D_m_*(*t*)/(*M*(*M* − 1)). To allow comparison with the real mitochondrial network, the total number of connections of the random network at each time point remains the same as that of the real network. In such a network, most mitochondria have approximately the same number of links close to the average number of links at time *t*, ∑ *_m_D_m_* (*t*)/*M* (Barabasi and Oltvai, 2004).

### Statistics

Wavelet analysis, correlation analysis, and fitting routines were performed using Matlab v7.6.0.324 (R2008a). Further statistics were performed using OriginPro 8 SR0 v8.0724 (B724).

## Results

### Inter-mitochondrial “mean field” correlation

**Figure 2:**
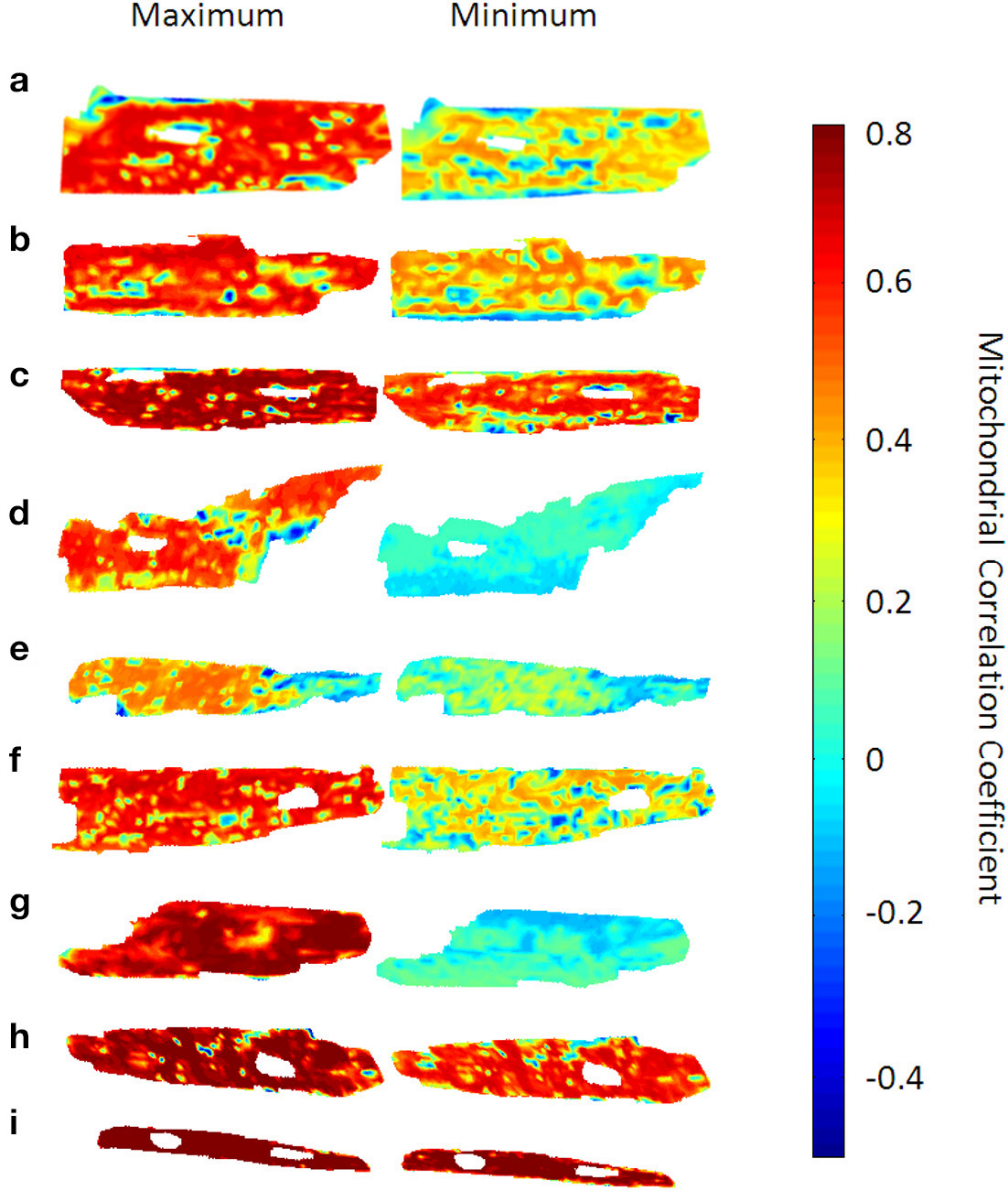
**Time-dependent mitochondrial “mean field” correlation maps for 9 cardiac myocytes**. The left hand side shows the correlation maps at the time point with the highest network mean correlation *c*(*t*) (see main text), whereas the right hand side shows those maps at the time point with the lowest mean correlation. Mitochondrial mean correlation values range between −0.4 and 0.8, the time-averaged mean correlation coefficient was found to be 0.43 ± 0.07. Also, some myocytes [myocytes (d) and (g)] show a prominent difference in overall correlation, while myocyte (i) does not show too pronounced correlation variability.

Mean-field coupling in complex non-linear biological networks of weakly coupled oscillators, such as a network of mitochondrial oscillators (Aon et al., 2006) or in other systems of coupled chemical or physical oscillators, assumes a global, all-to-all-coupling with a respective mean coupling constant (Kiss et al., 2002; Rougemont and Naef, 2006). In analogy to such a “mean field” approach, inter-mitochondrial correlation properties were examined at time *t* through signal correlations *c_i_*^(m)^ (*t*) of individual mitochondrion *m* with each of its *N_m_* topological network neighbors *m_i_* (Figure 2). The average correlation coefficient ∑ *_i_c_i_*^(m)^ (*t*)/*N_m_* for each mitochondrion *m* was then again averaged over all network constituents to determine the overall dynamic inter-mitochondrial mean correlation *c*(*t*) as *c*(*t*) = ∑ _*m*_ ∑ *_i_c_i_*^(m)^(*t*)/*N_m_*.

For each myocyte, correlation maps were created to visualize the distribution of average correlation coefficients *c_i_*^(m)^(*t*) within the myocyte at time *t*. To achieve this, values of *c_i_*^(m)^(*t*) were placed at the pixel-positions of the respective mitochondrion and missing inter-mitochondrial pixels were interpolated using the “griddata” function in Matlab v7.6.0.324 (R2008a). In Figure 2, only the time points with maximum and minimum values of *c*(*t*) for each respective myocyte are represented. It can be seen that, for some myocytes with maximum averaged correlation over all mitochondria, individual mitochondrial correlation values close to 80% can encompass almost the entire myocyte whereas the same can be valid for values of the correlation coefficient close to 0 (e.g., mitochondrion (d) and (g) in Figure 2). Averaged over all myocytes (*N* = 9) and time points, the mean correlation coefficient was found to be 0.43 ± 0.07.

### Mitochondrial network clustering

**Figure 3:**
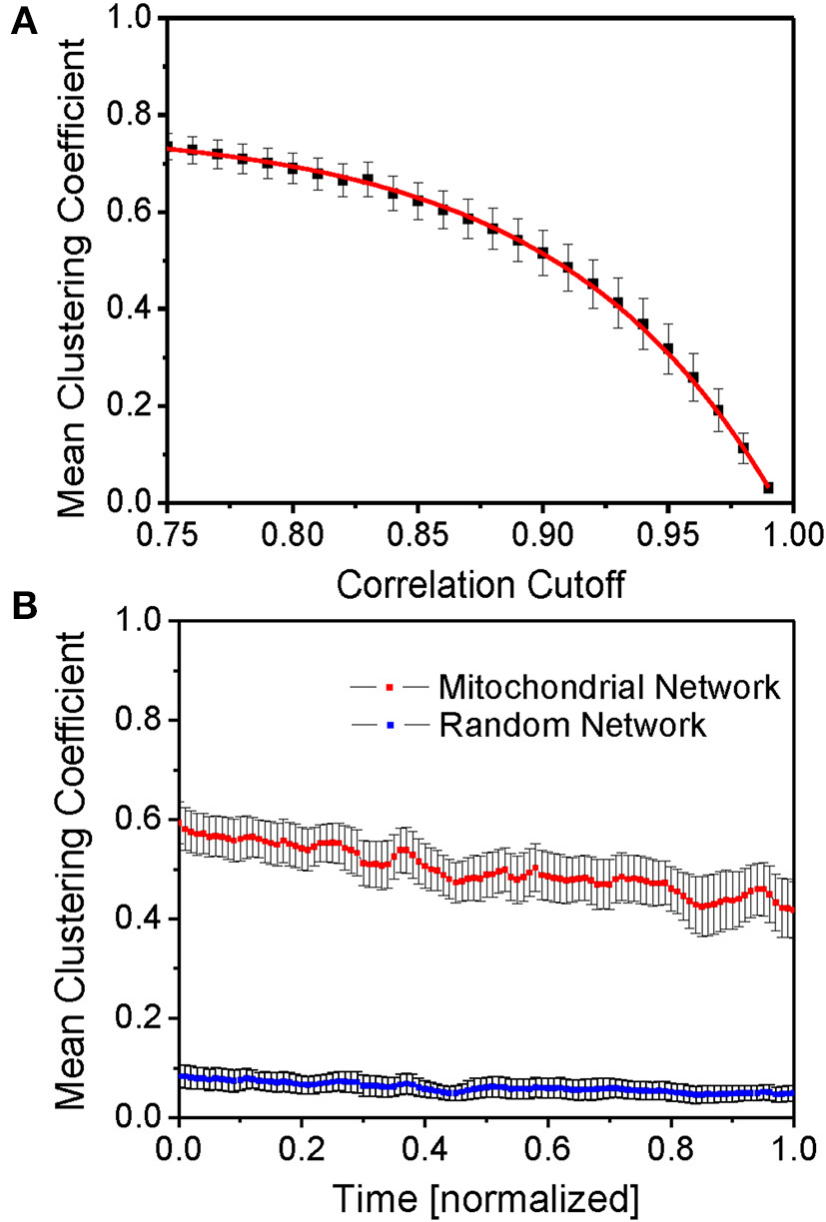
**Mean function clustering coefficient. (A)** Time- and myocyte-averaged mean clustering coefficient for different cutoffs of the correlation coefficient as the defining threshold of functional connectedness. The red curve is an exponential fit curve of the form *f*(*t*) = a · Exp [−*t/b*] + *c* (for details and fit parameters see main text). **(B)** Time evolution of the mean clustering coefficient averaged over different myocytes (*N* = 9) (red line) and for random networks on the same set of vertices and maximum degree (blue line). Evidently, the mitochondrial network, as opposed to the randomly constructed network, shows significant functional clustering.

The mean functional inter-mitochondrial clustering coefficient was determined for different correlation cutoffs (see Figure 3A). Naturally, high correlation coefficient cutoffs only involve few functionally connected mitochondria whereas lower cutoffs involve a higher number of mitochondria that are thought of as being connected. Therefore, the mean clustering coefficient increases for lower cutoffs. The ensuing fitted curve *f* (red line in Figure 3A, based on equation: *f*(*t*) = a · Exp [−*t/b*] + *c*) approximately crosses the 50% value of the mean clustering coefficients at correlation coefficients of 90% which also approximately corresponds to the median derivative value of the fitted curve. The fitting curve function parameters were determined as *a* = −7.81· 10^−6^ ± 1.33· 10^−6^, *b* = −8.64· 10^−2^ ± 0.13· 10^−2^ and *c* = 77.65· 10^−2^ ± 0.35· 10^−2^. Accordingly, and throughout the paper, two mitochondria are thought of as being connected, if their correlation exceeds 90%.

Time-normalization enables an estimate of the time evolution of the mean clustering coefficient (averaged over all mitochondria in the respective myocyte) for myocytes with unequal recording durations, and its comparison with that of random networks consisting of the same number of topological vertices and undirected links as the mitochondrial network of the respective myocyte (Figure 3B). Here, time-averaged mean clustering coefficients were found to be 0.500 ± 0.051 vs. 0.061 ± 0.020 for random networks, respectively.

### Major cluster properties in relation to mean mitochondrial clustering

**Figure 4:**
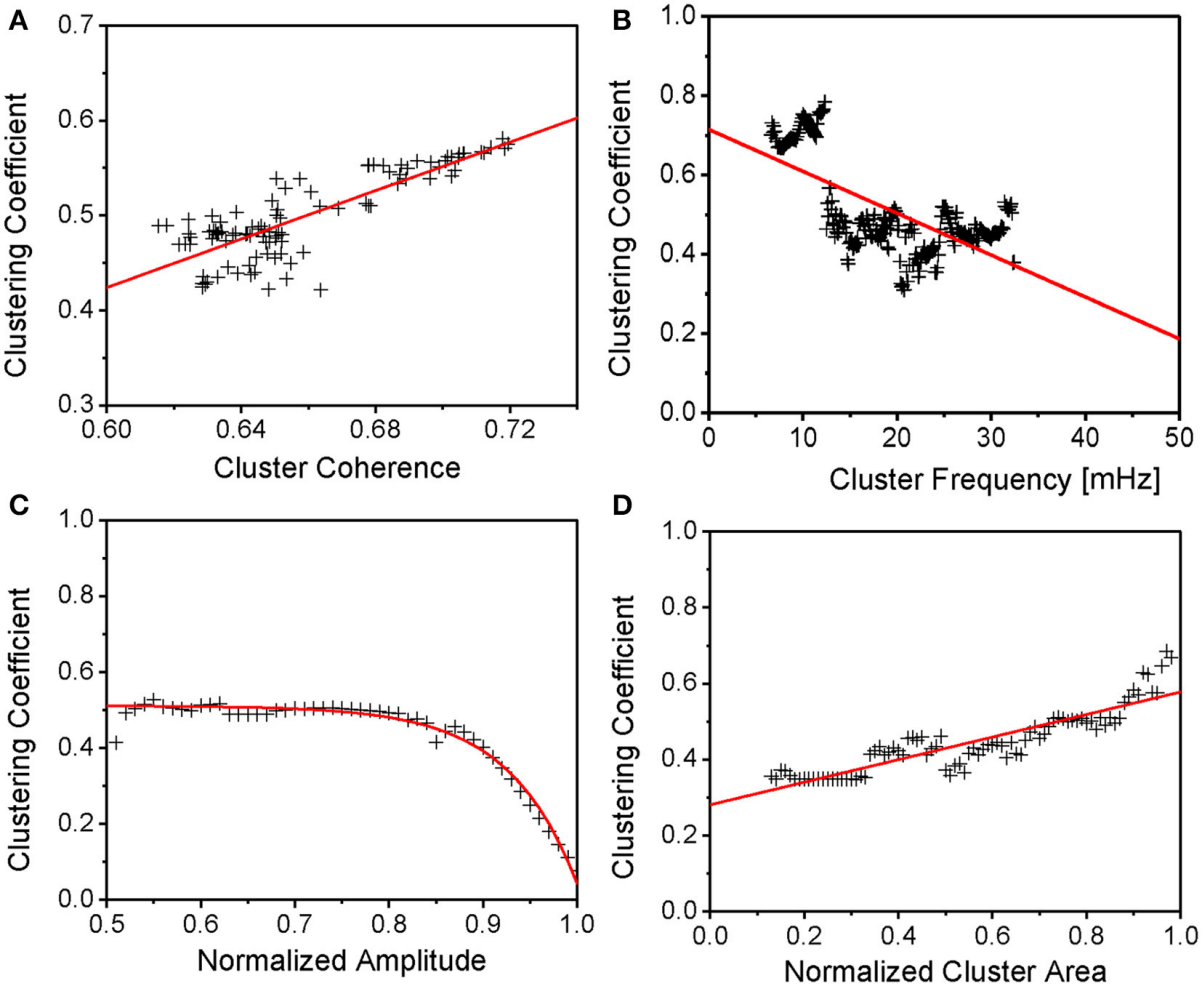
**Major Cluster Properties in Relation to Mean Mitochondrial Clustering. (A)** Mean clustering coefficient vs. major cluster coherence. Though cluster coherence variability is limited, mean clustering still increases naturally for increasing cluster coherence. **(B,D)** Mean clustering coefficient vs. major cluster frequency and normalized cluster area. Clustering is shown to increase with cluster area and, therefore, number of cluster mitochondria. In analogy to Figure 2 in Kurz et al. (2010a), clustering then decreases with increasing cluster frequencies. **(C)** Mean clustering coefficient vs. normalized cluster amplitude. Clustering reaches a plateau for normalized amplitudes below about 4/5 of the maximum amplitude, whereas clustering decreases toward zero for maximum amplitudes. The latter effect might be explained by a more prolonged and incomplete repolarization in between oscillations when the relative size of the cluster increases whereas the plateau might signify a maximum of mitochondrial clustering. However, further amplitude decrease might be due to a failure of the mitochondrial network to reenergize.

Individual mitochondrial signals are non-stationary in time and, therefore, wavelet transforms provide adequate means to examine the mitochondrion's signal temporal evolution (Grossmann et al., 1985). Following a recently described methodology (Kurz et al., 2010a), mitochondria were sorted according to the dynamic behavior of their frequencies such that clusters of mitochondria with similar frequencies could be identified. Mitochondria from the major frequency cluster were subsequently sampled and their mean temporal cluster area, amplitude and coherence determined (Kurz et al., 2010a). These spatio-temporal mitochondrial network properties are related to the functional topological network properties through the mean time-dependent clustering coefficient (see Figure 4).

In Figure 4A, it is shown that the mean clustering coefficient increases with increasing mitochondrial cluster coherence. The latter takes values between 0 and 1 at each frequency and measures the degree to which two signals are synchronous or correlated: a coherence of 1 is equivalent to perfect synchronization. It is assumed that mitochondrial clusters possess a high dynamic stability since their temporal coherence does not change significantly during the recording (Kurz et al., 2010a). The mean slope of the linear fit (red line in Figure 4A) was found to be 1.28 ± 0.09 with the interception point at −0.34 ± 0.06. This is not surprising since functional connectedness (as defined in the methods section) was chosen such that mitochondria are highly correlated. Higher degrees of clustering therefore correspond to a higher number of an individual mitochondrion's topological neighbors whose signals are highly correlated.

Furthermore, mitochondrial cluster frequency and normalized cluster area (see methods section) were compared against mean clustering coefficients (Figures 4B,D): larger cluster areas show a higher degree of functional clustering due to the fact that an increase in mitochondria with similar frequencies naturally increases the number of mitochondria that are functionally connected. The mean increase of the mean clustering coefficient versus normalized mitochondrial cluster area (in the form of the linear fit in Figure 4D, red line) was found as 0.30 ± 0.01, the interception point at 0.28 ± 0.01. Also, the mean clustering coefficient was considered against the mean mitochondrial cluster frequency (Figure 4B) and it can be seen that clusters with increasing ΔΨ*_m_* frequencies show lower clustering. The mean decrease of clustering versus major cluster frequency is −10.57· 10^−2^ ± 7.32· 10^−2^%/mHz (the interception point of the linear fit at 0 mHz being 0.71 ± 0.02). The result is in agreement with prior results (Kurz et al., 2010a) showing that higher cluster frequencies coincide with smaller normalized cluster area, thus, with Figure 4D, lower mean mitochondrial clustering coefficients (compare Figure 4D with Figure 2 in Kurz et al., 2010a).

Figure 4C presents the relation of topological clustering and the major cluster's amplitude, normalized to the maximum amplitude. Here, topological clustering reaches a plateau close to the value of the time-averaged mean clustering coefficient of 50% for oscillation amplitudes that are lower than 80% of the maximum amplitude of the major cluster. But, for amplitude values close to the maximum value, clustering decreases to minimal values. The linear fit in Figure 4C has been achieved with *f*(*t*) = a · Exp [−*t/b*] + *c* (as in Figure 3A)—fit parameters were found as *a* = −52.01 · 10^−8^ ± 26.97 · 10^−8^, *b* = −7.29 · 10^−2^ ± 0.28 · 10^−2^, and *c* = 51.10 · 10^−2^ ± 0.31 · 10^−2^. This is in agreement with prior observations (Kurz et al., 2010a) where it was shown that an increase in mitochondrial cluster amplitude corresponds to lower normalized cluster area, therefore, lower mean clustering coefficients (Figure 4 in Kurz et al., 2010a).

## Discussion

Mitochondrial networks in cardiac myocytes show evidence of interdependence between network structure and mitochondrial metabolism (Zorzano et al., 2010), as well as properties of a complex non-linear system under pathophysiological conditions (Aon, 2010). However, the only existing quantitative analysis of the mitochondrial network's topology is based on mitochondrial reticulum models (Sukhorukov et al., 2012), whereas other models mostly considered the networks biochemical properties (Wu et al., 2007; Zhou et al., 2010). Thus, there has been no prior study of the networks functional connectedness through quantitative individual mitochondrial signal analysis.

In the present work, we have shown that the mean correlation and the mean functional clustering coefficients in mitochondrial networks are spatially and temporally variable. The latter were shown to be significantly higher than those for random mitochondrial networks with the same respective number of mitochondria and inter-mitochondrial topological connections. Furthermore, topological network properties were related to the spatio-temporal properties of the major mitochondrial cluster with similar cluster coherence, frequency, normalized amplitude and cluster area. It could be shown that clustering increases with the percentage area of the cluster as well as the cluster's coherence. At first sight, this seems intuitively correct, however, the concept of clustering should not be confused with the notion of mitochondrial clusters in frequency. Nevertheless, increasing the number of mitochondria with similar frequencies, as is the case in synchronizing mitochondrial networks (Aon et al., 2006), increases the number of mitochondria in the network that are functionally linked, and, therefore, mean mitochondrial clustering increases. Also, in agreement with previous results (Kurz et al., 2010a), mean mitochondrial clustering was shown to decrease with higher frequencies supporting the notion that smaller clusters of mitochondria oscillate with a higher frequency. It has been proposed that this process can be due to temporal limitations of diffusion-mediated inter-mitochondrial coupling that starts in small nuclei of excited mitochondria (Aon et al., 2006) and progresses within the coupling medium (Kurz et al., 2010a), therefore leading to a decrease in the mean cluster frequency through an increase in depolarization time, and/or due to coupled mitochondrial oscillators adjusting to a common oscillatory mode with larger clusters taking longer to synchronize.

Additionally, it has been demonstrated that increased normalized cluster amplitudes are related to a decrease in mean mitochondrial clustering. This less intuitive result can be explained by the fact that mitochondria in the major cluster show a more prolonged as well as incomplete repolarization in between oscillations when the relative size of the cluster increases. Then again, the increase of cluster-incorporated mitochondria leads to an increase of topological clustering (cf. Aon et al., 2008b). Yet, Figure 4C indicates that this effect comes to a halt for amplitudes below approximately 4/5 of the maximum amplitude. In this amplitude realm, mean mitochondrial clustering remains constant at its time-averaged value for decreasing cluster amplitudes suggesting a stable maximum of topological connectedness. However, it should be noted that the effect of fluorescence intensity loss can be due to limitations of the TMRE readout that might be inadequate to track correlations in the state when redox equivalents supply is insufficient to reenergize the membrane. Under these conditions, energetic/redox impairment may eventually lead to cell death.

Mitochondrial networks consist of regularly organized lattice-like mitochondria that are seen as metabolic network hubs with multiple connections to anabolic and catabolic cellular pathways (Aon et al., 2008b; Aon and Cortassa, 2012). Their coordination was first dynamically examined in cardiac myocytes that were subjected to metabolic stress (Romashko et al., 1998; Aon et al., 2003) where it was presumed that ROS-induced ROS release acts as a coupling mediator between mitochondria to coordinate local and global cell-wide mitochondrial network behavior (Zorov et al., 2000; Aon et al., 2004). The concept of scale-free networks and its application to the mitochondrial network's functional connectivity has been the focus of recent studies (Aon et al., 2004, 2006, 2007, 2008b; Barabasi and Oltvai, 2004), which support the idea that the mitochondrial network architecture is connected to its function (Viola et al., 2009) and that metabolic supply changes can be due to morphological changes of the mitochondrial network structure, and, specifically, the morphology of mitochondrial membranes (Dimmer and Scorrano, 2006; Mcbride et al., 2006).

The present study also supports this concept in showing that topological clustering in mitochondrial networks is significant and relates directly to the network's spatio-temporal organization. It has also been shown that a mitochondrion's localization within the mitochondrial network has an effect on its functional properties (Lesnefsky et al., 1997; Kuznetsov et al., 1998). This is in agreement with the observation of strongly variable areas of mean-field correlation in mitochondrial networks as well as time-variant mitochondrial clustering. In addition, several studies could demonstrate that some individual mitochondria show independent responses to mitochondrial network dynamics (Loew et al., 1993; Duchen et al., 1998), again emphasizing mitochondrial functional heterogeneity and the complexity of the network's functional connectedness. The principle of functional connectedness applied in this study has also been used to describe brain functional networks that were shown to exhibit significantly larger clustering coefficients than those of random networks (Eguiluz et al., 2005); they are subject to intensive clinical research (see Bullmore and Sporns, 2009 for a review). Interestingly, correlations between spontaneous changes in brain activity generate very robust functional networks on timescales of seconds to minutes, i.e., in the same range as mitochondrial network dynamics (Greicius et al., 2003; Fox et al., 2005). Continuing this comparison, neuronal phase synchronization between coupled neurons was found to be highly dependent on clustering (Percha et al., 2005) and to possess self-organized critical dynamic properties (Siri et al., 2007).

The present analysis is limited to a functional analysis of the mitochondrial network. Furthermore, the present study only accounts for spatio-temporal properties of mitochondria in the major frequency clusters but neglects smaller clusters. This can be justified since the major cluster usually comprises the majority of network mitochondria that are above a percolation threshold of approximately 60% for lattice-like organized networks (cf. Aon et al., 2004). Nevertheless, the effect of long-range connections between different clusters and, therefore, the modularity of the network, will be the subject of further studies. Methods that examine the effective connectivity of the system to evaluate the causal influence of each system element on the network's behavior (see Friston, 2011 for a review), could be used to assess the connectivity between mitochondrial clusters within the myocyte or between neighboring myocytes, and consequently to examine scaling effects of mitochondrial clustering. It should also be noted that only two-dimensional slices of the cardiac myocytes have been examined here, that consequently contained only parts of the three-dimensionally arranged mitochondria. However, we would expect lateral coordination among rows of mitochondria above and below those in the focal plane to be similar to the communication between adjacent rows in the 2D plane, since the mitochondrial network has a symmetrical quasi-square lattice organization. The main source of anisotropy is the lateral versus longitudinal orientation of the mitochondria arrangement between the myofilaments, as evidenced by preferential depolarization propagation in the mitochondrial network along the axis parallel to the cardiac myocyte myofilaments (Kurz et al., 2010a).

In summary, the results of this study indicate that cardiac mitochondria constitute a collection of coupled network components that are subordinate to dynamic changes in their non-random functional connectedness.

### Conflict of interest statement

The authors declare that the research was conducted in the absence of any commercial or financial relationships that could be construed as a potential conflict of interest.
